# Procedural specificity in laparoscopic simulator training: protocol for a randomised educational superiority trial

**DOI:** 10.1186/1472-6920-14-215

**Published:** 2014-10-10

**Authors:** Flemming Bjerrum, Jette Led Sorensen, Lars Konge, Jane Lindschou, Susanne Rosthøj, Bent Ottesen, Jeanett Strandbygaard

**Affiliations:** Department of Obstetrics and Gynecology, The Juliane Marie Centre for Children, Women and Reproduction, Rigshospitalet, University of Copenhagen, Copenhagen, Denmark; Centre for Clinical Education, Simulationscenter Rigshospitalet, Capital Region of Denmark, Copenhagen, Denmark; Copenhagen Trial Unit, Centre for Clinical Intervention Research, Rigshospitalet, University of Copenhagen, Copenhagen, Denmark; Section of Biostatistics, Institute of Public Health, University of Copenhagen, Copenhagen, Denmark

**Keywords:** Laparoscopy, Specificity, Procedural training, Simulation, Virtual reality training

## Abstract

**Background:**

The use of structured curricula for minimally invasive surgery training is becoming increasingly popular. However, many laparoscopic training programs still use basic skills and isolated task training, despite increasing evidence to support the use of training models with higher functional resemblance, such as whole procedural modules. In contrast to basic skills training, procedural training involves several cognitive skills such as elements of planning, movement integration, and how to avoid adverse events. The objective of this trial is to investigate the specificity of procedural practice in laparoscopic simulator training.

**Methods/Design:**

A randomised single-centre educational superiority trial. Participants are 96 surgical novices (medical students) without prior laparoscopic experience. Participants start by practicing a series of basic skills tasks to a predefined proficiency level on a virtual reality laparoscopy simulator. Upon reaching proficiency, the participants are randomised to either the intervention group, which practices two procedures (an appendectomy followed by a salpingectomy) or to the control group, practicing only one procedure (a salpingectomy) on the simulator. 1:1 central randomisation is used and participants are stratified by sex and time to complete the basic skills. Data collection is done at a surgical skills centre.

The primary outcome is the number of repetitions required to reach a predefined proficiency level on the salpingectomy module. The secondary outcome is the total training time to proficiency. The improvement in motor skills and effect on cognitive load are also explored.

**Discussion:**

The results of this trial might provide new knowledge on how the technical part of surgical training curricula should be comprised in the future. To examine the specificity of practice in procedural simulator training is of great importance in order to develop more comprehensive surgical curricula.

**Trial registration:**

ClinicalTrials.gov: NCT02069951

## Background

Laparoscopic simulation training has become important in the acquisition of laparoscopic skills before operating on patients. Skills are ideally acquired through a structured curriculum using both feedback and predefined training goals [1]. Although there is a consensus that the curriculum should include a technical skills component, few studies have been conducted on exactly which technical skills should be included [2, 3]. Currently, there are only a few procedural modules for laparoscopy with solid evidence of the validity of metrics, including definition of proficiency levels [4]. In addition, many training curricula focus on isolated task training and do not incorporate procedural training [5]. Complex skills such as procedural training differ from isolated task training, as they involve more cognitive elements like planning, decision-making, and integration of isolated skills. When learning more complex skills in laparoscopic surgery, practicing only basic skills has not proven to be an effective strategy [6].

However, some of the elements practiced during procedural training may be transferable between different procedures. Thus, it is relevant to examine if repeated procedural simulator practice is relevant, which in turn will help develop the most sustainable curricula for minimally invasive surgical skills. Previous research have shown contradictory results; while some studies have found that laparoscopic skills are generalisable, other studies have found a high degree of task specificity for procedural training [7–9].

The objective of this trial is to examine the specificity of proficiency-based procedural simulator training and to examine transferability between two different procedural modules. Our hypothesis is that the same cognitive processes are utilised in the practicing of different complex laparoscopic tasks, like procedural training, and therefore these processes are transferable between different tasks.

## Methods and design

### Design

The trial is a randomised single-centre educational superiority trial.

### Participants

Participants are medical students recruited through an invitation distributed to student associations for general surgery and gynaecology and through a student newspaper. Inclusion criteria are: (1) enrolled at the Faculty of Health Science at the University of Copenhagen, (2) a bachelor’s degree in medicine, and (3) signed informed consent. Exclusion criteria are: (1) having previously participated in a trial involving laparoscopic training, (2) having performed laparoscopic surgery (>0 procedures), and (3) not speaking Danish at a conversational level. Every participant receives a unique trial identification number upon enrolment.

### Intervention

**Figure 1:**
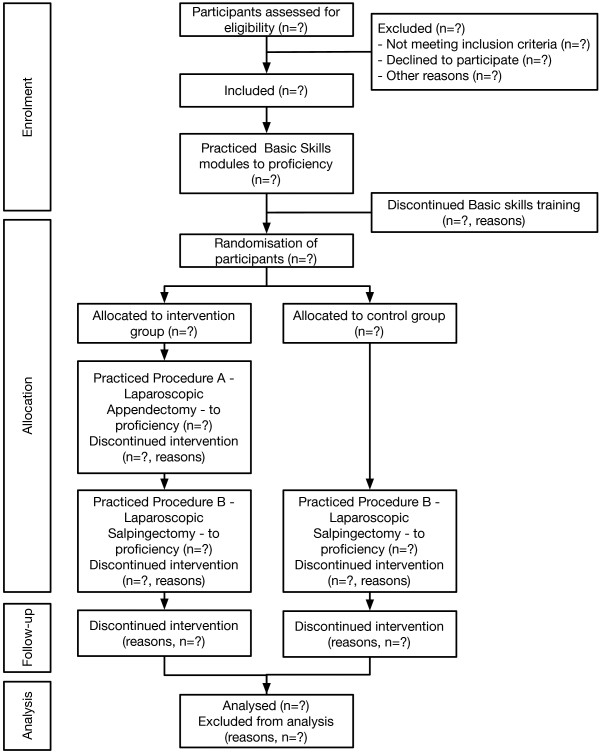
**Participant flowchart in accordance with the CONSORT statement.**

Participants are invited to participate in an introductory meeting in which information on the trial is given. All participants are informed verbally and in writing by the principal investigator before giving written informed consent. Participants can book three-hour training sessions through an online booking system; only one training session per day is permitted. Training sessions take place at the surgical skills centre at Rigshospitalet, University of Copenhagen. At the first training session, the participants are introduced to the simulator equipment by the principal investigator, and instructed on how to use it. The principal investigator supervises all training sessions and observes that the participants use both correct operating technique and handle instruments correctly. Participants start by practicing six basic skills tasks for isolated skills until they reach a predefined proficiency level for all tasks. The basic skills tasks do not have to be passed in any specific order. After each attempt, the simulator shows the participants automated feedback on each of the different measured parameters. Further more, the principal investigator provides feedback on request. The performance parameters and the predefined proficiency levels for each basic skill modules are listed in Table 1. Upon reaching proficiency for all tasks, the participants are randomised. Those randomised to the intervention group practice two procedures on the simulator to a predefined proficiency level: a laparoscopic appendectomy performed using endoloop technique (procedure A) and followed by a laparoscopic salpingectomy due to an ectopic pregnancy (procedure B). The control group only practices procedure B, Figure 1. The performance parameters and requirements for the predefined proficiency levels for procedures A and B are listed in Table 2. When practicing the two procedural modules, instructor feedback is provided after the first and tenth repetition [10]. The feedback is standardised and focuses on correct technique, instrument handling, and use of diathermy. For both the basic skills modules and the procedural modules, written instructions and video examples of the tasks are available for the participants and can be used at their own discretion. The predefined proficiency level for each module is reached when all of the requirements for the performance parameters is met simultaneously. This has to be archived for two attempts within five consecutive repetitions. The predefined proficiency levels are based on previous studies by the research group [1, 11]. Each participant is expected to use between six and twelve hours within a period of two months to complete the training program.

**Table 1:** **Performance parameters and requirements for the basic skills modules**

	Requirements for proficiency level for basic skills modules (maximum values)					
Performance parameters	1: Coordination	2: Instrument navigation	3: Grasping	4: Lifting and grasping	5: Fine dissection	6: Cutting
Total time (s)	150	-	-	120	150	200
Right instrument path length (m)	3	1.4	2	3.2	-	1.8
Right instrument angular path (degrees)	750	250	300	600	-	400
Right instrument time (s)	-	25	45	-	-	-
Right instrument misses (#/%)	-	2	-	60	-	-
Right instrument outside of view	4	-	-	-	-	-
Grasper collided with right box (#)	-	-	-	10	-	-
Right box lifted (#)	-	-	-	15	-	-
Left instrument path lenght (m)	0.8	1.4	2	3.2	-	2
Left instrument angular path (degrees)	300	250	300	600	-	400
Left instrument time (s)	-	25	45	-	-	-
Left instrument misses (#)	-	2	-	60	-	-
Left instrument outside of view	-	-	-	-	-	-
Grasper collided with left box (#)	-	-	-	10	-	-
Left box lifted (#)	-	-	-	15	-	-
Tissue damage (#)	5	-	3	5	-	10
Maximum damage (mm)	10	10	5	15	-	25
Misses (%)	12	-	-	-	-	-
Ripped or burned blood vessels (%)	-	-	-	-	0	-
Energy damaged on blood vessels (%)	-	-	-	-	20	-
Ripped small vessels (%)	-	-	-	-	25	-
Burned small blood vessels w/o stretch (%)	-	-	-	-	25	-
Rip failure (%)	-	-	-	-	-	25
Drop failure (%)	-	-	-	-	-	25
Max stretch damage	-	-	-	-	-	100

- : Not measure for the specific exercise.

**Table 2:** **Performance parameters and requirements for procedural modules A and B- : not measured for the specific module**

	Requirements for proficiency level for procedural modules (maximum values)	
Performance parameters	Procedure A: Appendectomy	Procedure B: Salpingectomy
Total time (s)	290	280
Right instrument path length (m)	5	3
Right instrument angular path (degrees)	710	450
Left instrument path length (m)	4	2
Left instrument angular path (degrees)	930	350
Pressure damage to appendix (#)	3	-
Appendix Grasping time (s)	3	-
Burn damage to cecum (#)	7	-
Distal loop correctly applied (Y/N)	Y	-
Proximal loops correctly applied (Y/N)	Y	-
Appendix divided	Y	-
Blood loss (ml)	-	180
Pool of blood (ml)	-	10
Ovary Diathermy damage (s)	-	3
Tube Cut: Uterus distance (mm)	-	3
Removed dissected tissue (Yes/No)	-	Y
Bleeding vessel cut (Yes/No)	-	Y

- : Not measured for the specific module.

### Simulation equipment

Four simulator stations, each with the virtual reality simulator LapSim® (Software Version 2013) from Surgical Science (Gothenburg, Sweden), are used. The station’s height is adjustable and consists of a 27” monitor, which is attached to a gaming computer with a pair of Simball 4D Joystick from G-coder Systems (Gothenburg, Sweden). All computers are connected to a local server containing an electronic database storing the data generated after each attempt on the simulator. Training stations are separated using wall dividers and there is a pair of noise cancelling Bose Quiet Comfort III earphones at each station. This reduces possible distractions and prevents participants from observing and interacting with each other during practice sessions.

### Randomisation

Copenhagen Trial Unit is responsible for a central computerised 1:1 randomisation. The allocation sequence is computer-generated, with a varying block size kept concealed from the investigator. A web-based response system is used to allocate participants after they have completed the basic skills modules (see Intervention). The stratification variables are sex (male/female) and training time to reach proficiency for all basic tasks (less than or equal to two hours/more than two hours).

### Blinding

Because of the nature of the intervention, it is not possible to blind the participants and the principal investigator to the allocation of the participants. Data entry is performed independently, without the involvement of the principal investigator. All forms are entered in a database using double data entry by external personal. A person other than the principal investigator, who is familiar with the simulator equipment, performs external data monitoring of the simulator data during the trial. The statistical analysis is blinded and the statistician is not aware of the intervention allocation of participants during the analysis; this is done with the two groups, coded as X and Y. After the analysis, two conclusions are drawn by the steering committee with the blinding intact; one conclusion assuming X is in the intervention group and Y is in the control group, and one conclusion assuming the opposite. Only thereafter is the randomisation code broken.

### Outcomes

The primary outcome is the number of repetitions necessary to reach the predefined proficiency level for procedure B. The secondary outcome is the total effective training time on the simulator for procedure B (in minutes). Exploratory outcomes are the cognitive load and parameters for movements, and time after the first attempt on procedure B. The cognitive load is measured using the Subjective Mental Effort Question (SMEQ), which is a cognitive workload questionnaire designed to allow individuals to rate the amount of effort invested during a task [12]. The questionnaire consists of a scale of 0 to 150 points, with nine scale markers with verbal statements ranging from ‘not at all hard to do’ to ‘tremendously hard to do’. The SMEQ has been previously been published and used in combination with simulator training [13, 14]. The SMEQ is filled out after the first attempt on procedure B. The performance parameters for movement (total path length for both right and left hand and total angular path for right and left hand) and time for the first attempt on procedure B are compared to assess the improvement in motor skills.

### Ethical considerations

The trial complies with the Helsinki Declaration on biomedical research and has been submitted to the Regional Scientific Ethics Committee, which found that in accordance with Danish regulations, ethical approval is not required for carrying out the trial (H-4-2013-FSP). The trial is registered at Clinicaltrials.gov (NCT02069951). The trial complies with the Consolidated Standards of Reporting Trials (CONSORT) statement for randomised trials (Figure 1) [15]. All participants receive verbal and written information on the trial before inclusion; participation is voluntary and participants do not receive any gifts or monetary compensation. All participants can withdraw from the trial at any time with no consequences to their future studies; participants are asked about their reasons for withdrawal but are not obliged to answer. All data is kept confidential and published anonymously.

### Sample size determination

Based on a previous study we expect the control group to take approximately 30 repetitions to reach the proficiency level, with a standard deviation of 15 [10]. A minimal difference of 10 repetitions between the two groups is deemed clinically relevant, meaning that the intervention group is expected to take approximately 20 repetitions to reach proficiency. The standard deviation is assumed to be equal in both groups. With alpha set to 0.05 and beta to 0.10 we need 48 participants in each group, totalling 96 participants.

### Statistical analysis

Data will be analysed using SPSS (Chicago IL, version 20.0), SAS statistical software (SAS Institute Inc., Cary NC, version 9.4) and the statistical software package R (version 3.0.3). A two-sided significance level of 0.05 will be used. The primary and secondary outcomes will be compared using the normal linear multivariable model, adjusting for the two stratification variables. If the assumptions of normality and homogeneity of variance are not sufficiently fulfilled, the outcome data will be transformed. If the assumptions cannot be met by transformation either robust regression, weighted least squares, or bootstrap methods will be applied. If none of these methods seem to give an adequate description of the data, the intervention and control group will be compared using the van Elteren test, and a non-parametric estimate of the confidence interval of the intervention effect will be obtained by bootstrapping [16, 17]. The p-values obtained for secondary and exploratory outcomes will be adjusted for multiplicity using the Benjamin-Hochberg procedure [18].

### Missing data

All analyses are performed according to the intention-to-treat principle. In case of less than 5 percent missing data for the randomised participants for primary and secondary outcome, a complete case analysis will be performed. In case of more than 5 percent missing data, a sensitivity analysis will be performed using a best-worst and worst-case scenario imputation. If these analyses allow for the same conclusion as the complete case analysis, the complete case analysis will be reported. If the best-worst and worst-best case analyses result in a different conclusion than the complete case analysis, multiple imputations will be performed. The multiple imputations will be based on the fully observed variables (that is, sex, intervention, SMEQ-score).

## Discussion

### Deliberate practice and task specificity

The presented trial design examines the transferability of skills between different procedural modules on a laparoscopic virtual reality simulator. A previous trial has found that training a basic skill is not an effective strategy for learning a more complex laparoscopic task [6], though most surgical training programs use only isolated or basic skills training [5, 19, 20]. Limited research has investigated which tasks and technical skills should be included in comprehensive minimally invasive surgical curricula [2]. Previous studies have focused on the importance of fidelity for transfer to a clinical setting or transferability between basic and more complex tasks. So far, no studies have examined the transferability between two complex tasks in laparoscopic training; thus, it is unclear whether it is necessary to practice more than one procedural module on a simulator. It is possible that the cognitive aspects of procedural training like instrument coordination, procedural planning, and error recognition could be taught using procedural modules that are different from the procedure that the trainees are supposed to perform in clinical practice. This concept is known as *positive transfer*, in which a previously practiced skill positively influences the acquisition of a new skill. The transfer effect can be caused by either similarities in the cognitive processes used to perform the skill or because the two skills contain many identical elements [21]. According to the specificity of practice hypothesis and Ericsson’s framework for deliberate practice the use of task specific training is important [21, 22]. This includes using both task-specific performance goals and a training setup resembling where the practiced skills is going to be used [23]. This is consistent with two randomised trials’ findings that using a model with higher functional resemblance may yield a better end result and increase transferability [7, 24, 25]. The same observation has been made in sports; for example, different types of throws exist in badminton and there may be a high degree of specificity when learning a specific skill or type of movement [21, 26, 27]. In contrast, results from a recent randomised trial found that practice using simple basic skills compared with a nephrectomy module gave better results when performing a VATS lobectomy in a simulated setting [9]. This is surprising, since the nephrectomy module is more similar to a VATS lobectomy in terms of task resemblance.

A randomised trial has shown that, compared to no training at all, practicing a laparoscopic cholecystectomy on a virtual reality simulator resulted in improved performance when practicing a laparoscopic nephrectomy in a porcine model [8]. This shows that some degree of skills-transfer is seen between different laparoscopic procedures. Furthermore, previous trials have focused on examining the effect in a single-test setting, not considering the effect on the learning process for reaching proficiency for a skill. Developing procedural modules for virtual reality training is very challenging and time-consuming, and it is therefore essential to determine whether it is necessary to develop training modules for many procedures.

### Cognitive load

Increased cognitive load can negatively influence the learning of a new skill and simulator training can be used the to reduce the cognitive load and help facilitate the learning process [28]. Through practicing a procedure on a simulator, the surgical novice can become familiar with the procedure, learn critical steps and how to avoid possible adverse events. For novices, the cognitive load associated with learning a new procedure gradually decreases with continuous simulator practice [14]. Whether this reduction in cognitive load is generalizable and still present when learning a new procedure is unknown and has not been previously examined.

### Trial strengths and limitations

The strength of the present trial is the use of a trial design that mirrors an actual curriculum, because it incorporates both proficiency-based training and distributed practice [29]. Therefore, the findings can probably be applied to actual training in simulation centres. The use of basic skills training to ensure the same proficiency level before randomisation helps standardise the intervention with the procedural modules because the participants have a similar starting point in terms of basic laparoscopic skills. Additionally, use of the same training equipment minimises the risk of confounding from variations in training conditions.

The trial is designed in order to minimise the risks of systematic errors and the risks of random errors [30–32]. Systematic errors have been sought to be reduced by central randomisation stratified for prognostic factors [30–32]. Blinding is used whenever possible and data is analysed according to the intention-to-treat principle. We are aware that some outcomes are at risk of being assessed with some bias, as they are not possible to blind [30–32].

A limitation is the trial participants are medical students instead of novice surgical residents; however, previous studies have shown that medical students’ performance on the simulator is similar to that of novice surgical residents [10, 11]. There are only approximately 50 first-year residents in surgery and gynaecology in the region every year, and they vary greatly in laparoscopic experience; thus, the trial would not have been feasible with first-year residents. Ideally, the trial should include transfer to the clinical settings using clinical outcomes, but this is not feasible due to the large sample size that is required and the difficulty with finding relevant clinical outcomes [33].

### Trial status

Currently, participants are being included and randomisation is still ongoing. Data collection is expected to end in September of 2014.
